# Relationship between weight-adjusted waist circumference index and prevalence of gallstones in U.S. adults: a study based on the NHANES 2017-2020

**DOI:** 10.3389/fendo.2023.1276465

**Published:** 2023-10-27

**Authors:** Bin Ke, Ying Sun, Xin Dai, Yang Gui, Song Chen

**Affiliations:** ^1^ Department of Gastrointestinal Surgery, The Second People’s Hospital of Wuhu City (Affiliated Wuhu Hospital of East China Normal University), Wuhu, China; ^2^ Department of Nursing, The Second People’s Hospital of Wuhu City (Affiliated Wuhu Hospital of East China Normal University), Wuhu, China; ^3^ Department of Gastrointestinal Surgery, The Affliated Chuzhou Hospital of Anhui Medical University (The First People’s Hospital of Chuzhou), Chuzhou, China

**Keywords:** gallstones, WWI index, cross-sectional study, metabolic syndrome, NHANES (National Health and Nutrition Examination Survey)

## Abstract

**Objective:**

We aimed to assess the association between weight-adjusted waist circumference index (WWI) and gallstone prevalence in US adults.

**Methods:**

We selected individuals from the National Health and Nutrition Examination Survey (NHANES) database from 2017 to 2020 and used logistic regression analyses, subgroup analyses, and dose-response curves to assess the association between WWI and gallbladder stone prevalence and age, sex, and ethnicity.

**Results:**

A total of 7971 participants aged ≥20 years were enrolled in our study; 828 patients had a self-reported history of gallstones. After correcting for confounders, for each unit of WWI after Ln conversion, the prevalence of gallbladder stones increased by 34% (OR=1.34, 95% CI:1.20, 1.50). Dose-response curves showed a positive correlation between WWI and gallbladder stone prevalence.According to the subgroup analysis, the positive association between TyG index and high-frequency HI was more significant in males(OR=1.34, 95% CI:1.07, 1.69), <40 years old(OR=1.42, 95% CI:1.18, 1.71), white people Americans(OR=1.35, 95% CI:1.08, 1.68) and other races(OR= 1.56, 95% CI:1.13, 2.14).

**Conclusion:**

Higher WWI was positively associated with the prevalence of gallbladder stones and was associated with gender, age, and ethnicity. This is noteworthy, although a causal relationship could not be established.

## Introduction

Cholelithiasis is a common global disease and one of the most common digestive disorders.Epidemiologic evidence suggests that approximately 5-25% of adults in the United States and Europe suffer from cholelithiasis (1, 2). Gallstones are a benign biliary tract disorder but are also a high risk factor for the development of gallbladder carcinoma (3, 4). The incidence of gallstones varies greatly among different ethnic groups in the world, with the incidence of gallstones in American Indians as high as 70%, and the prevalence of gallstones in adult Caucasians ranging from 10% to 15% (5, 6), compared to the relatively low prevalence of gallstones in Asian populations (7, 8). In general, the symptoms of gallstone disease are not obvious, but a small percentage of the population may develop serious complications (3, 9, 10). Although some risk factors for gallstone formation have been more or less reported in previous studies, there is a lack of reliable clinical indicators to further predict the risk of gallstone formation.

Obesity has always been a global health problem, a threat to public health and safety around the world, and is inextricably linked to the development of many diseases. One projected study suggests that for the United States, 50% of adults will be obese by 2030 (11). This sounds frightening, and the fact that the current obesity rate for adults in the U.S. has reached a staggering 40% tells us that this prediction is not alarmist (12). Obesity has now been shown to be strongly associated with the following diseases including, but not limited to, cardiovascular disease, hypertension, diabetes, hyperlipidemia, stroke, cancer, psychiatric disorders, and sexual functioning and gynecological problems, among others (13–16), Body Mass Index (BMI) is the most widely used parameter for evaluating obesity in recent days’ applications, yet This most common metric has the disadvantage that BMI is unable to differentiate between body fat and muscle mass and is affected by a variety of factors including age, gender, and racial differences (17, 18). In order to differentiate between the effects of waist circumference and body fat on body weight, Park et al. proposed a new obesity assessment metric, the Weight-Adjusted Waist Index (WWI) (19), which is the most widely used parameter for evaluating obesity. This index normalizes waist circumference (WC) to body weight, which is more reasonable and easier to measure than BMI alone. Several studies have shown a positive correlation between the WWI and mortality from new-onset hypertension, diabetes, and cardiovascular disease (20, 21).

Previous studies on cholelithiasis have shown that risk factors for gallstone formation generally include ethnicity, gender, pregnancy, and age over 40 years, with a corresponding 4- to 10-fold increase in the risk of developing gallstones (1, 2). The metabolic syndrome is the most important modifiable risk factor for cholelithiasis (22). Among these factors, obesity, especially abdominal adiposity, is strongly associated with the development of gallstones (22). The WWI has shown that obesity is closely related to the development of gallstones. Abdominal obesity is closely associated with the development of gallstones.Despite multiple evidences suggesting that obesity is inextricably linked to the development of gallstones, there is still a lack of reliable obesity-related indices for predicting and evaluating the risk of gallstones.The WWI has a higher sensitivity and specificity than traditional body parameters such as body mass index (BMI). We therefore hypothesized that there is a relationship between WWI and the occurrence of gallstones, and therefore, in this study, we aimed to evaluate the value of WWI in the occurrence of gallstones in the adult population in the United States.

## Materials and methods

### Research population

**Figure 1:**
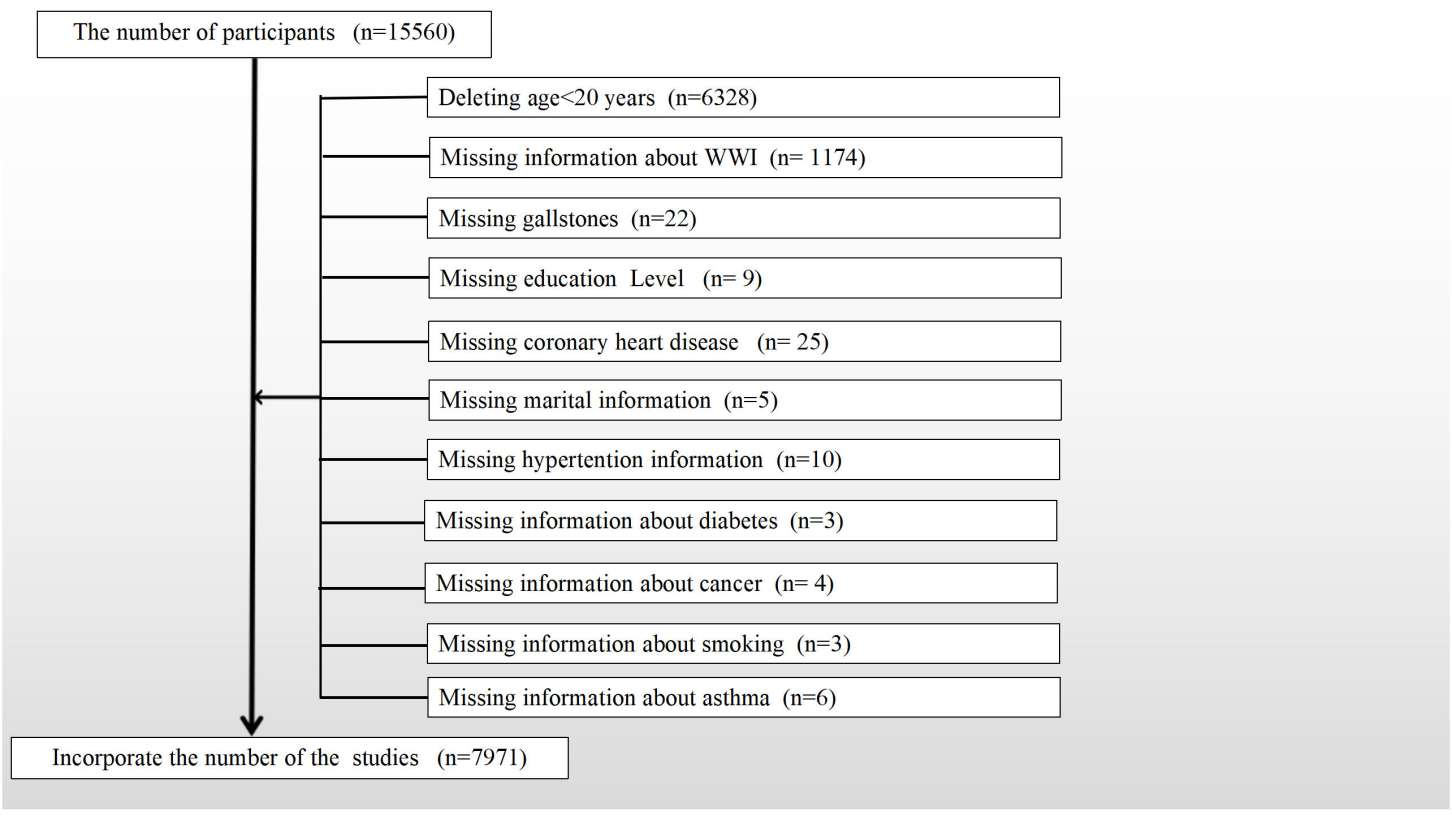
Flow chart for participants.

Data were obtained from NHANES, an open-source database officially maintained by the CDC in the U.S. NHANES is a cross-sectional survey that has been updated every two years for the past 20 years, with a population of about 10,000 people at a time, and only about 60% of the population was surveyed and completed in 2019-2020 due to the New Crown Epidemic. The year 2017-2020 was used for the study data because the questionnaire for gallstones was only available during that time period. Since the questionnaire was only administered to adults aged 20 years and older, we removed participants under the age of 20 years, and based on the purpose of our study, we screened the study population, with detailed inclusion and exclusion criteria provided in **Figure 1**. In conclusion, a final total of 7971 cases were included in this study, including 828 who self-reported a history of gallstones.

### Data collection

WWI index was designed as an exposure variable.WWI=Waist circumference (cm)/square root weight (kg) (23). Triglyceride and fasting glucose levels were determined enzymatically using an automated biochemical analyzer. Serum triglyceride concentrations were determined using a Roche Cobas 6000 chemistry analyzer and a Roche Modular P. The serum triglyceride levels were measured using a Roche Cobas 6000 chemistry analyzer. The prevalence of gallstones was assessed by the following questionnaire, including “Have you ever been told you have gallstones? The occurrence of gallstones was used as an outcome variable. Potential covariates that could confound the association between WWI and gallstones were summarized in multivariate adjustment models. Covariates in our study included: sex (male/female), age (years), race categorized as Mexican American, white people, black people, and other (22), education level (categorized as less than high school level, high school, and more than high school level), poverty-to-income ratio (PIR) shifted to a categorical variable in accordance with previous studies (22), marital status (married or with a partner unmarried), alcohol consumption (from questionnaire ALQ101-Had at least 12 alcohol drinks/1 yr? Participants who answered yes were identified as alcohol drinkers), physical activity, cholesterol level (mg/dl), smoking status (based on questionnaire SMQ020-Smoked at least 100 cigarettes in life, participants who answered yes were considered smokers), hypertension, diabetes mellitus, coronary heart disease, cancer, and asthma (based on the questionnaire, participants who answered yes were identified as having these diseases). Considering also that insulin resistance is a risk factor for gallstone formation, we additionally adjusted the METS-IR index, which was found to be associated with an increased prevalence of gallstones (22). There were also dietary intake factors, including energy intake, fat intake, sugar intake, and water intake, and all participants were eligible for two 24-hour dietary recalls, and the average consumption of the two recalls will be used in our analysis.

### Handling of missing values

Continuous variables with a high number of missing values were converted to categorical variables, and missing variables were set as a dummy variable group and named “unclear”. All detailed measurement procedures using the study variables are publicly available at www.cdc.gov/nchs/nhanes/.

### Statistical methods

p<0.05 was considered statistically significant. Empower software and R4.2.0 were used for all analyses. The use of appropriate NHANES sampling weights for statistical analyses was recommended in the official website, and detailed guidelines for weight analysis were provided.New sampling weights for the combined survey cycle were constructed by dividing the 2-year weights for each cycle by 3.2 (24). The weights provided in the dataset were parsed using the survey design R package in R. Since the study employs sampling weights in the analysis, Design-Based Wald Test of Association were used. Continuous variables are expressed as weighted survey means and 95% CIs, while categorical variables are expressed as weighted survey and 95% CIs.Following the STROBE guidelines, we constructed three multivariate regression models. In Model 1, no adjustment for covariates was made. Model 2 adjusted for gender, age, race, education level, and marital status. Model 3 adjusts for all variables. We also used generalized additive modeling (GAM) and smooth curve fitting to address the nonlinearity of WWI with gallstones. If nonlinear correlations were observed, a two-band linear regression model (segmented regression model) was used to fit each interval and calculate threshold effects. In sensitivity analyses, the WWI was converted from a continuous variable to a bicategorical variable to assess its robustness. Linear trend tests were conducted using the two quartiles of WWI as categorical variables. The accuracy of the results was further verified using propensity scores (nearest neighbor matching method with the caliper value set at 0.01 for 1:1 matching and matching variables including gender, age, and race) and inverse probability weighting. Subgroup analyses by sex, age, and race were also performed by stratified multiple regression analysis. In addition, interaction terms were added to test for heterogeneity of associations between subgroups using a log-likelihood ratio test model.

## Results

### Baseline characteristics of the participants

The baseline demographic characteristics of the included participants are shown in **Table 1**. A total of 7971 participants were included in this study, of which 3898 (48.9%) were male. Weighted characteristics were categorized according to the presence or absence of gallstones. There were significant differences in baseline characteristics between the two groups except for education, cholesterol level, and asthma prevalence status. Individuals with gallstones were more likely to have higher age and WWI levels, had a significantly higher prevalence of females, and had more diabetes, hypertension, and coronary heart disease.

**Table 1:** Baselines characteristics of participants,weighted.

Characteristic	Non-stone formers	Stone formers	P-value
Age(years)	47.14 (46.05,48.23)	56.85 (55.62,58.09)	<0.0001
WWI Index	10.98 (10.93,11.03)	11.48 (11.40,11.56)	<0.0001
Gender(%)			<0.0001
Male	51.18 (49.47,52.90)	26.77 (22.84,31.10)	
Female	48.82 (47.10,50.53)	73.23 (68.90,77.16)	
Race(%)			0.0036
Mexican American	15.74 (12.81,19.18)	14.81 (11.29,19.20)	
white people	62.55 (57.26,67.55)	69.47 (62.64,75.53)	
black people	11.75 (9.00,15.20)	7.09 (5.25,9.51)	
Other Race	9.97 (8.02,12.32)	8.63 (5.83,12.59)	
Education Level(%)			0.1164
Less than high school	10.57 (9.43,11.82)	9.56 (7.11,12.75)	
High school	26.55 (23.71,29.60)	31.39 (26.53,36.68)	
More than high school	62.88 (59.28,66.35)	59.05 (54.53,63.42)	
Marital Status(%)			0.0001
Cohabitation	62.37 (59.65,65.01)	64.55 (59.05,69.69)	
Solitude	17.59 (16.18,19.10)	23.20 (19.26,27.66)	
Alcohol(%)			<0.0001
Yes	13.96 (12.70,15.33)	26.62 (21.94,31.89)	
No	76.22 (74.45,77.91)	62.38 (58.05,66.53)	
Unclear	9.81 (8.48,11.33)	10.99 (8.28,14.46)	
High Blood Pressure(%)			<0.0001
Yes	30.25 (27.94,32.67)	49.29 (43.25,55.36)	
No	69.75 (67.33,72.06)	50.71 (44.64,56.75)	
Diabetes(%)			<0.0001
Yes	10.11 (9.25,11.05)	20.05 (17.24,23.18)	
No	89.89 (88.95,90.75)	79.95 (76.82,82.76)	
Asthma(%)			0.1432
Yes	14.99 (13.65,16.44)	17.89 (14.04,22.51)	
No	85.01 (83.56,86.35)	82.11 (77.49,85.96)	
Coronary Artery Disease(%)			<0.0001
Yes	3.64 (2.52,5.22)	7.29 (5.29,9.96)	
No	96.36 (94.78,97.48)	92.71 (90.04,94.71)	
Cancers(%)			<0.0001
Yes	10.25 (9.32,11.27)	18.52 (14.35,23.57)	
No	89.75 (88.73,90.68)	81.48 (76.43,85.65)	
Smoked(%)			0.0342
Yes	42.07 (40.05,44.12)	47.81 (41.17,54.52)	
No	57.93 (55.88,59.95)	52.19 (45.48,58.83)	
Physical Activity(%)			<0.0001
Never	49.52 (47.66,51.39)	33.47 (28.75,38.53)	
Moderate	27.93 (25.77,30.20)	36.57 (31.10,42.41)	
Vigorous	22.54 (21.22,23.92)	29.96 (25.59,34.73)	
PIR(%)			0.0009
<1.3	16.47 (15.11,17.93)	15.72 (12.03,20.27)	
≥1.3<3.5	30.06 (27.33,32.94)	39.78 (32.98,46.99)	
≥3.5	42.34 (39.31,45.43)	35.59 (31.06,40.38)	
Unclear	11.13 (9.53,12.96)	8.92 (6.55,12.04)	
Serum Cholesterol(%)			0.89
Lower	45.68 (42.94,48.44)	44.87 (39.33,50.54)	
Higher	48.83 (46.10,51.55)	50.00 (43.94,56.06)	
Unclear	5.49 (4.52,6.66)	5.13 (3.43,7.60)	
METS-IR(%)			0.0014
Lower	49.04 (47.12,50.97)	39.90 (34.97,45.05)	
Higher	45.41 (43.43,47.40)	54.97 (49.40,60.42)	
Unclear	5.54 (4.56,6.73)	5.13 (3.43,7.60)	

For continuous variables: survey-weighted mean (95% CI), P-value was by survey-weighted linear regression (svyglm).

For categorical variables: survey-weighted percentage (95% CI), P-value was by survey-weighted Chi-square test (svytable).

### A higher WWI index was associated with a higher prevalence of gallstones

**Figure 2:**
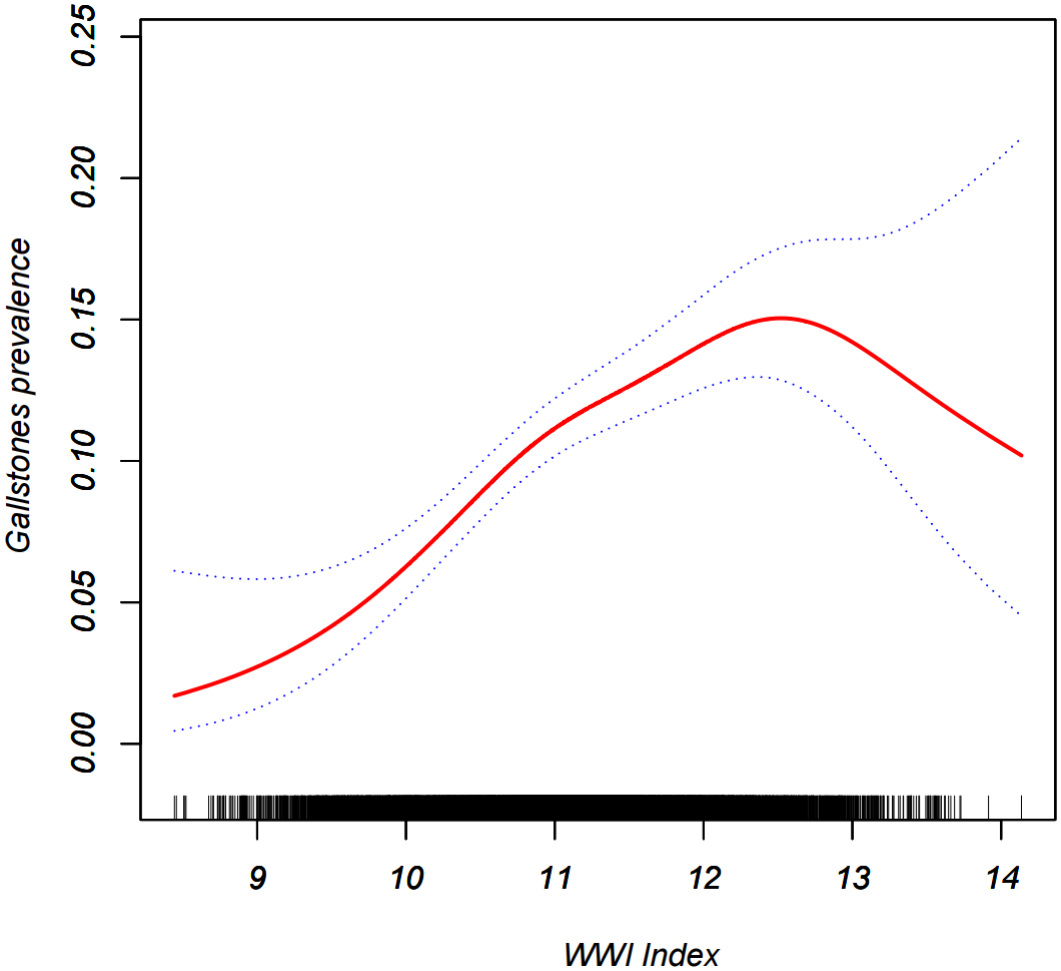
Density dose-response relationship between WWI index with gallstones prevalence.The area between the upper and lower dashed lines is represented as 95% CI. Each point shows the magnitude of the WWI index and is connected to form a continuous line.Adjusted for all covariates except effect modifier.

Multiple regression analyses with different adjustments for the effect of confounders on the correlation showed that a positive association between the WWI index and the prevalence of gallstones was detected in both the crude and the minimal/fully adjusted models. In the fully adjusted model, each unit increase in the WWI index was associated with a 34% increased risk of gallstones prevalence (OR=1.34, 95% CI:1.20, 1.50) (**Table 2**). Generalized additive modeling and smoothed curve fitting were used to further explore the WWI index and gallstone prevalence. Our results showed a nonlinear positive correlation between the WWI index and gallstones (**Figure 2**). The likelihood ratio test revealed a threshold effect for the effect of WWI index with gallstone prevalence, with an optimal inflection point value at 10.71 (**Table 3**).

**Table 2:** Logistic regression analysis between WWI index with gallstones prevalence.

Characteristic	Model 1 OR (95%CI)	Model 2 OR (95%CI)	Model 3 OR (95%CI)	Model 4 OR (95%CI)
WWI	1.99 (1.82, 2.17)	1.50 (1.35, 1.66)	1.34 (1.20, 1.50)	1.36 (1.19, 1.57) △
Categories				
Lower	1	1	1	1
Higher	2.65 (2.26, 3.10)	1.68 (1.41, 2.00)	1.36 (1.23, 1.51)	1.32 (1.08, 1.63) ★
P for trend	<0.01	<0.01	<0.01	<0.01

Model 1 was adjusted for no covariates;

Model 2 was adjusted for age, gender, race, marital status and education;

Model 3 was adjusted for covariates in Model 2+diabetes, blood pressure, PIR, total water, total kcal, total sugar, total fat, smoked, physical activity, alcohol use, serum cholesterol, METS-IR serum creatinine, coronary artery disease, asthma and cancers were adjusted.

Model 4 adjusts for the same covariates as Model 3.

△= PSM analysis only in model 4.

★ = IPTW analysis only in model 4.

**Table 3:** Two-piecewise linear regression and logarithmic likelihood ratio test explained the threshold effect analysis of WWI index with gallstones prevalence.

WWI Index	ULR Test	PLR Test	LRT test
	OR(95%CI)	OR(95%CI)	P value
<10.71	1.34 (1.20, 1.50)	2.70 (1.73, 4.20)	<0.0001
≥10.71		1.18 (1.03, 1.35)	

ULR, univariate linear regression; PLR, piecewise linear regression; LRT, logarithmic likelihood ratio test, statistically significant:p<0.05.

### Sensitivity analyses

Results after further 1:1 PSM analysis showed that each unit increase in WWI index was associated with a 36% increase in the risk of gallstones (OR=1.36, 95% CI:1.19, 1.57) (**Table 2**, **Supplementary Table 1**). When the WWI index was converted to dichotomous, logistic regression revealed that a significant 36% increase in the risk of gallstone prevalence was observed in the highest group compared to the lowest WWI index group (OR=1.36,95% CI:1.23, 1.51. P for trend<0.01). Further we constructed an inverse probability weighted analysis of the results after dichotomizing the WWI and **Supplementary Table 2** shows that baseline information no longer differed between the two groups, and the inverse probability weighted logistic regression showed that a significantly increased risk of gallstone prevalence of 32% was observed in the highest group compared to the lowest WWI-indexed group (OR=1.32,95% CI:1.08, 1.63) (**Table 2**; **Supplementary Table 2**).

### Subgroup analysis

Subgroup analyses were performed to assess the robustness of the association between the WWI index and gallstone prevalence. Results indicated significant odds ratios (OR) in demographic groups: 1.34 (95% CI: 1.07, 1.69) for males, 1.33 (95% CI: 1.17, 1.51) for females, 1.64 (95% CI: 1.27, 2.10) for individuals under 40 years of age, 1.42 (95% CI: 1.18, 1.71) for those aged 40 to 59, and 1.16 (95% CI: 0.99, 1.37) for individuals aged 60 and over. Additionally, within different racial and ethnic groups, the odds ratios were as follows: 1.15 (95% CI: 0.90, 1.47) for Mexican Americans, 1.44 (95% CI: 1.21, 1.72) for white individuals, 1.35 (95% CI: 1.08, 1.68) for black individuals, and 1.56 (95% CI: 1.13, 2.14) for other ethnic groups. We also tested for interactions with sex, age, and race. No correlations were detected for interaction p-values that met statistical significance, except for age, **Table 4**.

**Table 4:** Subgroup regression analysis between WWI index with gallstones prevalence.

Characteristic	Model 1	Model 2	Model 3	P for interaction ★
	OR (95%CI)	OR (95%CI)	OR (95%CI)	
Stratified by gender				0.41
Male	2.16 (1.81, 2.58)	1.51 (1.22, 1.88)	1.34 (1.07, 1.69)	
Female	1.69 (1.53, 1.88)	1.47 (1.30, 1.65)	1.33 (1.17, 1.51)	
Stratified by age(years)				0.0009
20-39	2.33 (1.89, 2.88)	1.90 (1.52, 2.37)	1.64 (1.27, 2.10)	
40-59	1.90 (1.61, 2.23)	1.63 (1.38, 1.94)	1.42 (1.18, 1.71)	
60-85	1.53 (1.32, 1.76)	1.31 (1.12, 1.53)	1.16 (0.99, 1.37)	
Stratified by race				0.95
Mexican American	1.84 (1.52, 2.23)	1.23 (0.98, 1.55)	1.15 (0.90, 1.47)	
white people	2.01 (1.74, 2.32)	1.60 (1.36, 1.87)	1.44 (1.21, 1.72)	
black people	1.86 (1.56, 2.22)	1.51 (1.24, 1.85)	1.35 (1.08, 1.68)	
Other Race	2.07 (1.61, 2.65)	1.67 (1.24, 2.23)	1.56 (1.13, 2.14)	

Model 1 was adjusted for no covariates;

Model 2 was adjusted for age, gender, race, marital status and education;

Mode3 = adjusted for all covariates except effect modifier.

★ = only in model 3.

## Discussion

Our study is the first comprehensive analysis of the association between WWI and gallstones in this large, prospective, nationally representative study of the U.S. population. A two-cycle population-based study (2017-2020) based on the NHANES database. Results showed that WWI was positively associated with gallstone prevalence, with each unit increase in the WWI index associated with a 34% increased risk of gallstone prevalence in the fully adjusted model (OR=1.34, 95% CI:1.20, 1.50). These results strongly support the value of WWI as a predictor of gallstone development.

The prevalence of cholelithiasis is high globally, with approximately 5-25% of adults suffering from cholelithiasis in the Western world alone (1), and 1.5 million people in the United States seeking medical attention for cholelithiasis-related conditions in 2015 alone (25). At the same time, the prevalence of obesity has risen dramatically, now reaching unprecedented levels: nearly one-third of the world’s population suffers from obesity (26). Gallstones are generally caused by a combination of factors, including genetic and environmental factors. The high risk of morbidity in obese patients is mainly due to the metabolic changes caused by obesity, with abnormalities occurring in various metabolic organs, including the liver and gallbladder, such as excessive bile secretion by the liver, hyperlipidemia, and reduced intestinal motility (27). In this study by interaction test we found that there is an effect of age on the prevalence of gallstones. In fact, it is still controversial whether age is a high-risk factor for gallstone incidence. Previous studies have reported that age is the main risk factor for gallstone incidence (28, 29), which is in agreement with our results, but there are still studies that show that other factors such as obesity (30) have a greater impact on the incidence of gallstones, especially in the younger population. As for gender, the call for female patients to have a higher risk of gallstone incidence seems to be higher (31, 32), our study report rejected this high risk of incidence due to gender difference, which may suggest that there is also a high risk of gallstone incidence in males, which suggests that we should not ignore the risk of incidence of gallstones in male patients. Certain genetic factors may also increase the risk of gallstone disease. In the United States, white people Americans and other races have the highest prevalence of gallstone disease, which may be related to differences in diet and lifestyle habits between races. The vast majority of black people Americans do not work under the same conditions as white people Americans, which means that white people Americans have more access to a better quality diet, which is likely to be is contributing to the higher incidence of gallstones. As society continues to develop, the prevalence is gradually increasing with the development and refinement of high-calorie foods as well as the increasing number of sedentary and physically inactive people (33, 34).

In our study, we used an integrated regression model to minimize the effect of confounding factors, ensure the stability of the results, and elucidate the association between the WWI index and the prevalence of gallstones. Our findings are generally consistent with the aforementioned studies. To investigate the stability of the results, we used propensity score and inverse probability weighting. Among them, propensity score has been widely used in observational studies (35). The results we obtained after pragmatic PSM remained in good agreement and were more useful. However, it is undeniable that PSM still has its own limitations (36), and as shown in the **Supplementary Table** the baseline information between the two groups after PSM still cannot eliminate the statistical differences. For this reason, we referred to the study of chen et al. (37) to introduce the inverse probability weighting method, which was used to eliminate the between-group differences in the baseline data, to further validate the stability of the WWI index on the prevalence of gallstones. The results remained stable after inverse probability weighting.

The specific mechanisms linking elevated WWI indices with increased prevalence of gallstones are unclear, but based on previous studies, there are a number of potential mechanisms that may exist.1. A central mechanism of obesity or metabolic syndrome is insulin resistance, which has been shown to be associated with the formation of gallbladder stones (22). Studies have shown that insulin resistance leads to the production of cholesterol-saturated bile in high-risk Hispanic populations, with altered gallbladder function as a result, leading to gallbladder stone formation (38). Animal studies have found that isolated hepatic insulin resistant mice (LIRKO mice) are more likely to form cholesterol gallstones (39). Mice given a high-protein and high-quality diet were found to form sludge and gallstones more quickly (40).2. Another factor involved in the pathogenesis of gallstones is leptin, which is involved in the development of hyperleptinemia when obesity occurs (41, 42). *In vivo* experiments confirmed that long-term intraperitoneal administration of high doses of leptin (10 μ/g/d) induced weight loss in food-fed C57BL/6J ob/ob mice was associated with cholesterol gallstone formation (43). In another *in vitro* experiment it was also demonstrated that leptin affects cholelithiasis formation by modulating bile acid metabolism (44).

Our study has several strengths. First, the NHANES study participants were a representative sample from the U.S. who strictly followed a well-designed study protocol and underwent rigorous quality control and assurance to ensure that our conclusions were reliable. Secondly NHANES provides extensive demographic and metabolic data, as well as extensive follow-up with a median of over 23 years. This allowed us to adjust for major confounders of concern in our multivariate models. In addition we adjusted for confounding variables and performed subgroup analyses to ensure that our results were applicable to a wider range of individuals. However, our study also has some limitations. First, our study was a cross-sectional study and we were unable to elucidate the causal relationship between WWI and gallstones. Second, survey data from NHANES were based on questionnaires, which means that recall bias may exist. Despite these limitations, this paper still reveals for the first time the relationship between WWI and gallstone prevalence and provides strong support for WWI as a predictor of gallstone development.As a next step, we will conduct a multicentre prospective cohort study and construct relevant clinical prediction models to further explore the impact of the WWI index on the prevalence of gallstones in the real world.

## Conclusion

This study suggests that elevated WWI levels are associated with a higher likelihood of gallstone prevalence, and that assessing by WWI index levels may benefit gallbladder health, and that the benefit may be greater in young people in particular. However, further studies are still needed to validate our findings.

## Institutional review board statement

The NCHS Research Ethics Review Committee approved the NHANES survey protocol(https://www.cdc.gov/nchs/nhanes/irba98.htm), and all participants of the study provided informed written consent.The NHANES database is open to the public and therefore the ethical review of this study was exempt.

## Informed consent statement

Informed consent was obtained from all subjects involved in the study.

## Data availability statement

The raw data supporting the conclusions of this article will be made available by the authors, without undue reservation.

## Ethics statement

Written informed consent was obtained from the individual(s) for the publication of any potentially identifiable images or data included in this article.

## Author contributions

BK: Investigation, Software, Visualization, Writing – review & editing. YS: Investigation, Software, Visualization, Writing – review & editing. XD: Data curation, Software, Writing – original draft. YG: Data curation, Software, Writing – original draft. SC: Conceptualization, Methodology, Writing – original draft.

## Supplementary material

Supplementary Table 1 Comparison of baseline data for gallstone classification using Psm.

Supplementary Table 2 Comparison of baseline data for Wwi classification by using Iptw.
