# Supplementary material for: Relationship between weight-adjusted waist circumference index and prevalence of gallstones in U.S. adults: a study based on the NHANES 2017-2020
Source: Front Endocrinol (Lausanne). 2023 Oct 27;14:1276465. doi: 10.3389/fendo.2023.1276465 (PMC10641849; doi:10.3389/fendo.2023.1276465)
Supplement: Supplementary Table 1 — Comparison of baseline data for gallstone classification using Psm. [file Table_1.docx]

Supplementary Table 1. Comparison of baseline data for gallstone classification using PSM.

| Characteristic | Non-stone formers | Stone formers | P value |
| --- | --- | --- | --- |
| Age(years) | (828) 54.37 ±15.97 | (828) 57.78 ±15.77 | <0.0001 |
| WWI Index | (828) 11.36± 0.78 | (828) 11.55 ±0.76 | <0.0001 |
| Gender(%) |  |  | <0.0001 |
| Male | 368 (44.4) | 239 (28.9) |  |
| Female | 460 (55.6) | 589 (71.1) |  |
| Race(%) |  |  | <0.0001 |
| Mexican American | 420 (50.7) | 106 (12.8) |  |
| white people | 140 (16.9) | 350 (42.3) |  |
| black people | 58 (7) | 171 (20.7) |  |
| Other Race | 55 (6.6) | 107 (12.9) |  |
| Education Level(%) |  |  | <0.0001 |
| Less than high school | 298 (36) | 140 (16.9) |  |
| High school | 189 (22.8) | 217 (26.2) |  |
| More than high school | 341 (41.2) | 471 (56.9) |  |
| Marital Status(%) |  |  | 0.477 |
| Cohabitation | 516 (62.3) | 493 (59.5) |  |
| Solitude | 209 (25.2) | 229 (27.7) |  |
| Alcohol(%) |  |  | 0.0056 |
| Yes | 166 (20) | 221 (26.7) |  |
| No | 536 (64.7) | 497 (60) |  |
| Unclear | 126 (15.2) | 110 (13.3) |  |
| High Blood Pressure(%) |  |  | <0.0001 |
| Yes | 301 (36.4) | 452 (54.6) |  |
| No | 527 (63.6) | 376 (45.4) |  |
| Diabetes(%) |  |  | 0.0006 |
| Yes | 152 (18.4) | 211 (25.5) |  |
| No | 676 (81.6) | 617 (74.5) |  |
| Asthma(%) |  |  | <0.0001 |
| Yes | 104 (12.6) | 167 (20.2) |  |
| No | 724 (87.4) | 661 (79.8) |  |
| Coronary Artery Disease(%) |  |  | <0.0001 |
| Yes | 31 (3.7) | 71 (8.6) |  |
| No | 797 (96.3) | 757 (91.4) |  |
| Cancers(%) |  |  | <0.0001 |
| Yes | 75 (9.1) | 145 (17.5) |  |
| No | 753 (90.9) | 683 (82.5) |  |
| Smoked(%) |  |  | <0.0001 |
| Yes | 298 (36) | 381 (46) |  |
| No | 530 (64) | 447 (54) |  |
| Physical Activity(%) |  |  | 0.0001 |
| Never | 329 (39.7) | 261 (31.5) |  |
| Moderate | 201 (24.3) | 269 (32.5) |  |
| Vigorous | 298 (36) | 298 (36) |  |
| PIR(%) |  |  | 0.0293 |
| ＜1.3 | 217 (26.2) | 195 (23.6) |  |
| ≥1.3＜3.5 | 278 (33.6) | 319 (38.5) |  |
| ≥3.5 | 206 (24.9) | 219 (26.4) |  |
| Unclear | 127 (15.3) | 95 (11.5) |  |
| Total Kcal(%) |  |  | 0.0173 |
| Lower | 353 (42.6) | 393 (47.5) |  |
| Higher | 290 (35) | 294 (35.5) |  |
| Unclear | 185 (22.3) | 141 (17) |  |
| Total Sugar(%) |  |  | 0.0089 |
| Lower | 337 (40.7) | 333 (40.2) |  |
| Higher | 306 (37) | 354 (42.8) |  |
| Unclear | 185 (22.3) | 141 (17) |  |
| Total Fat(%) |  |  | 0.0239 |
| Lower | 350 (42.3) | 369 (44.6) |  |
| Higher | 293 (35.4) | 318 (38.4) |  |
| Unclear | 185 (22.3) | 141 (17) |  |
| Total Water(%) |  |  | 0.0064 |
| Lower | 322 (38.9) | 375 (45.3) |  |
| Higher | 321 (38.8) | 312 (37.7) |  |
| Unclear | 185 (22.3) | 141 (17) |  |
| Serum Creatinine |  |  | 0.028 |
| Lower | 469 (56.6) | 420 (50.7) |  |
| Higher | 318 (38.4) | 350 (42.3) |  |
| Unclear | 41 (5) | 58 (7) |  |
| METS-IR |  |  | 0.2409 |
| Lower | 311 (37.6) | 298 (36) |  |
| Higher | 475 (57.4) | 472 (57) |  |
| Unclear | 42 (5.1) | 58 (7) |  |
| Serum Cholesterol |  |  | 0.0064 |
| Lower | 351 (42.4) | 395 (47.7) |  |
| Higher | 436 (52.7) | 375 (45.3) |  |
| Unclear | 41 (5) | 58 (7) |  |

For continuous variables: (N) Mean ± SD, Standardized difference = abs(Mean1-Mean0)/sqrt((S1+S2)/2)

For categorical variables: N (%), Standardized difference = abs(P1-P0)/sqrt((P1*(1-P1)+P0*(1-P0))/2)

Supplementary Table 2.Comparison of baseline data for WWI classification by using IPTW.

| Variables | Lower | Higher | P value | P value |
| --- | --- | --- | --- | --- |
| Age(years) | 50.52 ± 17.15 | 50.97 ± 17.40 | 0.026 | 0.2512 |
| Gender(%) |  |  |  | 0.4736 |
| Male | 0.481 | 0.473 | 0.016 |  |
| Female | 0.519 | 0.527 | 0.016 |  |
| Race(%) |  |  |  | 0.9017 |
| Mexican American | 0.116 | 0.117 | 0.003 |  |
| white people | 0.34 | 0.348 | 0.015 |  |
| black people | 0.272 | 0.263 | 0.02 |  |
| Other Race | 0.171 | 0.173 | 0.007 |  |
| Education Level(%) |  |  |  | 0.9539 |
| Less than high school | 0.179 | 0.182 | 0.007 |  |
| High school | 0.247 | 0.246 | 0.002 |  |
| More than high school | 0.574 | 0.572 | 0.003 |  |
| Marital Status(%) |  |  |  | 0.6589 |
| Cohabitation | 0.59 | 0.587 | 0.006 |  |
| Solitude | 0.22 | 0.228 | 0.019 |  |
| Alcohol(%) |  |  |  | 0.9181 |
| Yes | 0.187 | 0.187 | 0.001 |  |
| No | 0.677 | 0.675 | 0.006 |  |
| Unclear | 0.135 | 0.139 | 0.009 |  |
| High Blood Pressure(%) |  |  |  | 0.8429 |
| Yes | 0.384 | 0.386 | 0.004 |  |
| No | 0.616 | 0.614 | 0.004 |  |
| Diabetes(%) |  |  |  | 0.3548 |
| Yes | 0.144 | 0.151 | 0.021 |  |
| No | 0.856 | 0.849 | 0.021 |  |
| Asthma(%) |  |  |  | 0.4022 |
| Yes | 0.17 | 0.163 | 0.019 |  |
| No | 0.83 | 0.837 | 0.019 |  |
| Coronary Artery Disease(%) |  |  |  | 0.9832 |
| Yes | 0.044 | 0.044 | 0 |  |
| No | 0.956 | 0.956 | 0 |  |
| Cancers(%) |  |  |  | 0.8625 |
| Yes | 0.105 | 0.104 | 0.004 |  |
| No | 0.895 | 0.896 | 0.004 |  |
| Smoked(%) |  |  |  | 0.5439 |
| Yes | 0.409 | 0.416 | 0.014 |  |
| No | 0.591 | 0.584 | 0.014 |  |
| Physical Activity(%) |  |  |  | 0.3303 |
| Never | 0.42 | 0.409 | 0.023 |  |
| Moderate | 0.278 | 0.293 | 0.033 |  |
| Vigorous | 0.301 | 0.298 | 0.008 |  |
| PIR(%) |  |  |  | 0.9642 |
| ＜1.3 | 0.239 | 0.24 | 0.002 |  |
| ≥1.3＜3.5 | 0.342 | 0.343 | 0.002 |  |
| ≥3.5 | 0.29 | 0.285 | 0.01 |  |
| Unclear | 0.129 | 0.132 | 0.008 |  |
| Total Kcal(%) |  |  |  | 0.859 |
| Lower | 0.405 | 0.41 | 0.012 |  |
| Higher | 0.397 | 0.392 | 0.01 |  |
| Unclear | 0.198 | 0.197 | 0.003 |  |
| Total Sugar(%) |  |  |  | 0.7732 |
| Lower | 0.394 | 0.401 | 0.016 |  |
| Higher | 0.408 | 0.401 | 0.013 |  |
| Unclear | 0.198 | 0.197 | 0.003 |  |
| Total Fat(%) |  |  |  | 0.9581 |
| Lower | 0.402 | 0.405 | 0.007 |  |
| Higher | 0.4 | 0.398 | 0.004 |  |
| Unclear | 0.198 | 0.197 | 0.003 |  |
| Total Water(%) |  |  |  | 0.9783 |
| Lower | 0.398 | 0.4 | 0.005 |  |
| Higher | 0.403 | 0.402 | 0.002 |  |
| Unclear | 0.198 | 0.197 | 0.003 |  |
| Serum Creatinine |  |  |  | 0.9177 |
| Lower | 0.462 | 0.467 | 0.009 |  |
| Higher | 0.471 | 0.466 | 0.008 |  |
| Unclear | 0.067 | 0.067 | 0.002 |  |
| METS-IR |  |  |  | 0.8884 |
| Lower | 0.468 | 0.463 | 0.01 |  |
| Higher | 0.464 | 0.47 | 0.011 |  |
| Unclear | 0.068 | 0.067 | 0.003 |  |
| Serum Cholesterol |  |  |  | 0.9798 |
| Lower | 0.449 | 0.448 | 0.003 |  |
| Higher | 0.484 | 0.486 | 0.004 |  |
| Unclear | 0.067 | 0.067 | 0.002 |  |

P-value were from weighted t test for continuous variables, and weighted chi-square test for categorical variables

For continuous variables, Standardized difference = abs(Mean1-Mean0)/sqrt((S1+S2)/2)

For categorical variables, Standardized difference = abs(P1-P0)/sqrt((P1*(1-P1)+P0*(1-P0))/2)
